# P22077 inhibits LPS-induced inflammatory response by promoting K48-linked ubiquitination and degradation of TRAF6

**DOI:** 10.18632/aging.103309

**Published:** 2020-06-09

**Authors:** Xi-Bao Zhao, Fei-Yang Ji, Hong-Rui Li, Hui-Hui Zhu, Zi-Zhao Zhao, Jing Ling, Qian-Qian Di, Xing-Yu Ma, Wei-Lin Chen

**Affiliations:** 1 Guangdong Provincial Key Laboratory of Regional Immunity and Diseases, Department of Immunology, Shenzhen University School of Medicine, Shenzhen 518060, China; 2 Institute of Immunology, Zhejiang University School of Medicine, Hangzhou 310006, China

**Keywords:** P22077, tumor necrosis factor receptor-associated factor 6, ubiquitin specific protease-7, inflammation, ubiquitination

## Abstract

Inflammation is a biological process associated with multiple human disorders such as autoimmune diseases and metabolic diseases. Therefore, alleviation of inflammation is important for disease prevention or treatment. Recently, deubiquitinating enzymes (DUBs), especially ubiquitin specific protease-7 (USP7) attracts increasing attention as a potential drug target for inflammation. As an inhibitor of USP7, P22077 has been used to study the roles of USP7 in inflammatory response and neuroblastoma growth. However, the role and precise mechanism of P22077 in anti-inflammatory is still indistinct. In this study, we demonstrated that P22077 could attenuate the release of pro-inflammatory factors including TNF-α, IL-1β, IL-6 and NO, suppress mRNA expression of COX-2 and iNOS, and inhibit activation of NF-κB and MAPKs signaling pathways in Raw264.7 cells and mouse peritoneal macrophages after LPS stimulation. *In vivo* study showed that P22077 could relieve inflammatory response and reduce the lung injury in C57BL/6 mice with LPS-induced endotoxemia. Mechanically, P22077 might play an anti-inflammatory role by promoting tumor necrosis factor receptor-associated factor 6 (TRAF6) degradation via K48-linked polyubiquitination. These findings provide a rationale for the role of the P22077 in anti-inflammatory pathway and the promising clinical application of P22077 to treat inflammatory diseases.

## INTRODUCTION

Inflammation is the body’s immediate response to tissue damage by pathogens or other stimuli. However, excessive inflammation contributes to inflammatory diseases such as rheumatoid arthritis (RA), metabolic diseases and cardiovascular diseases [1]. Macrophages play a critical role in the initiation of inflammation by releasing inflammatory mediators and pro-inflammatory cytokines. Macrophages recognize microorganisms via a limited number of pattern-recognition receptors (PRRs) including Toll-like receptors (TLRs), RIG-I-like receptors (RLRs), C-type lectin receptors (CLRs) et al. [2]. Lipopolysaccharide (LPS) is one of the TLR4 ligand and the signaling through TLR4 activates an intracellular signaling cascade that leads to nuclear translocation of transcription factors, such as activator protein-1 (AP-1) and Nuclear factor κB (NF-κB) [3–5]. Upon LPS stimulation, TLR4 adaptor protein MyD88 recruits interleukin 1 receptor associated kinase 1 (IRAK1) and interleukin 1 receptor associated kinase 4 (IRAK4), resulting in recruitment of tumor necrosis factor receptor-associated factor 6 (TRAF6), and then TRAF6 is activated by Lys63 (K63)-linked autoubiquitinated [6–8]. Activated TRAF6 recruits and activates TGF-beta-activated kinase 1 (TAK1), TGF-beta activated kinase 1 binding protein 1 (TAB1), and TGF-beta activated kinase 1 binding protein 2 (TAB2) complex [9–11]. Next, this complex induces activation of IKKs complex (consists of the IKKα, IKKβ and IKKγ), the activated IKKs complex catalyzes IκBs phosphorylation and leads IκBs degradation in proteasome manner, resulting in p65 activation and induction of inflammatory cytokines production [12, 13].

TRAF6 is an adapter protein with 570 amino acids that is broadly expressed in mammalian tissues and highly species conservative (consists of an N-terminal Zn RING finger domain, a series of five Zn finger domains, a coiled-coil TRAF-N domain and a C-terminal TRAF-C domain) [14, 15]. TRAF-C domain ensures its signaling specificity and Zn RING finger domain is essential for downstream activation of NF-κB [16, 17]. TRAF6 plays a pivotal role in TLR4-mediated signaling pathway. Usually, TRAF6 forms a homo-dimer and catalysts K63-linked ubiquitination. Recently it was reported that TRAF6 can also be modified by K48-linked ubiquitination and promoting TRAF6 proteasomal degradation to block the inflammatory process [14, 18].

Ubiquitination and deubiquitination are two reverse processes, as an important post-translational modification, like phosphorylation and dephosphorylation, play a crucial role in multiple physiological processes. Ubiquitination mediates proteins linked-polyubiquitin chains to target proteins, and deubiquitination removes polyubiquitin chains from target proteins. Deubiquitinating enzymes (DUBs) play a central role in regulating protein deubiquitination process which have more than eighty family members [19, 20]. Ubiquitin Specific Protease-7 (USP7) or herpesvirus-associated ubiquitin-specific protease (HAUSP) is a deubiquitinating enzyme which first been found as a tumor suppressor *in vivo* through stabilizing p53 [21]. Later, it was also found to regulate inflammation by deubiquitination of NF-κB signaling pathway proteins including NF-κB and NEMO [22, 23]. Importantly, knockdown USP7 expression in gastric epithelial cells coincided with reducing cellular TRAF6 and p53 proteins [24, 25], and these results suggested that USP7 could regulate TRAF6 protein stability. P22077 is an inhibitor of USP7 [26, 27], and it has been found attenuating the p53-dependent apoptotic pathway and inhibiting neuroblastoma growth [28, 29]. As TRAF6 is an important protein in inflammation process, we suppose that P22077 might regulate inflammation response by targeting TRAF6 protein.

In this study, we demonstrated that P22077 exerts significant anti-inflammatory effects *in vitro* and *in vivo* through inhibition of the NF-κB and MAPKs pathways via promoting K48-linked ubiquitination and degradation of TRAF6. These findings suggested that P22077 could be a promising agent for treatment inflammatory diseases.

## RESULTS

### P22077 does not affect cell viability of Raw264.7 cells and mouse peritoneal macrophages

**Figure 1:**
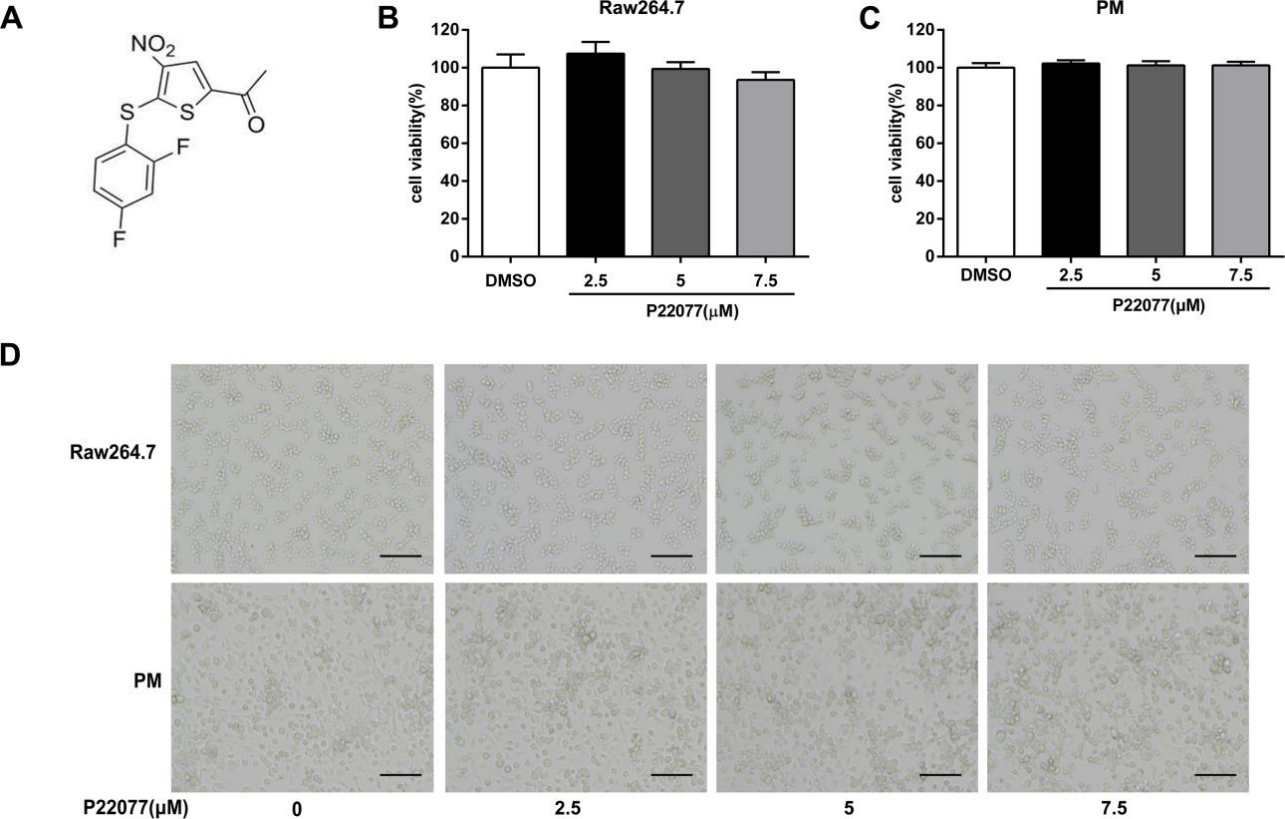
**P22077 does not affect cell viability.** (**A**) The chemical structure of P22077. (**B**, **C**) The cytotoxicity of P22077 in Raw264.7 cells (**B**) and mouse peritoneal macrophages (PM) (**C**). (**D**) Raw264.7 cells and peritoneal macrophages plated treated by different concentrations of P22077 for 12h, the morphology of the cells observed under the microscope. Scale bars, 100μm. Similar results were obtained from three independent experiments and data were presented as mean ± SD of one representative experiment.

The chemical structure of P22077 was shown in Figure 1A. To determine the cytotoxicity of P22077, MTT assay was used to evaluate the cell viability in macrophages. As shown in Figure 1B, 1C, P22077 (2.5, 5.0 and 7.5 μM) had no significant cytotoxicity both in Raw264.7 cells and mouse peritoneal macrophages. Similarly, no obvious changes were observed in cell density of P22077-treated Raw264.7 cells and mouse peritoneal macrophages (Figure 1D). Therefore, P22077 at these concentrations were selected for the subsequent cellular experiments.

### P22077 inhibits LPS-induced inflammatory response in Raw264.7 cells

**Figure 2:**
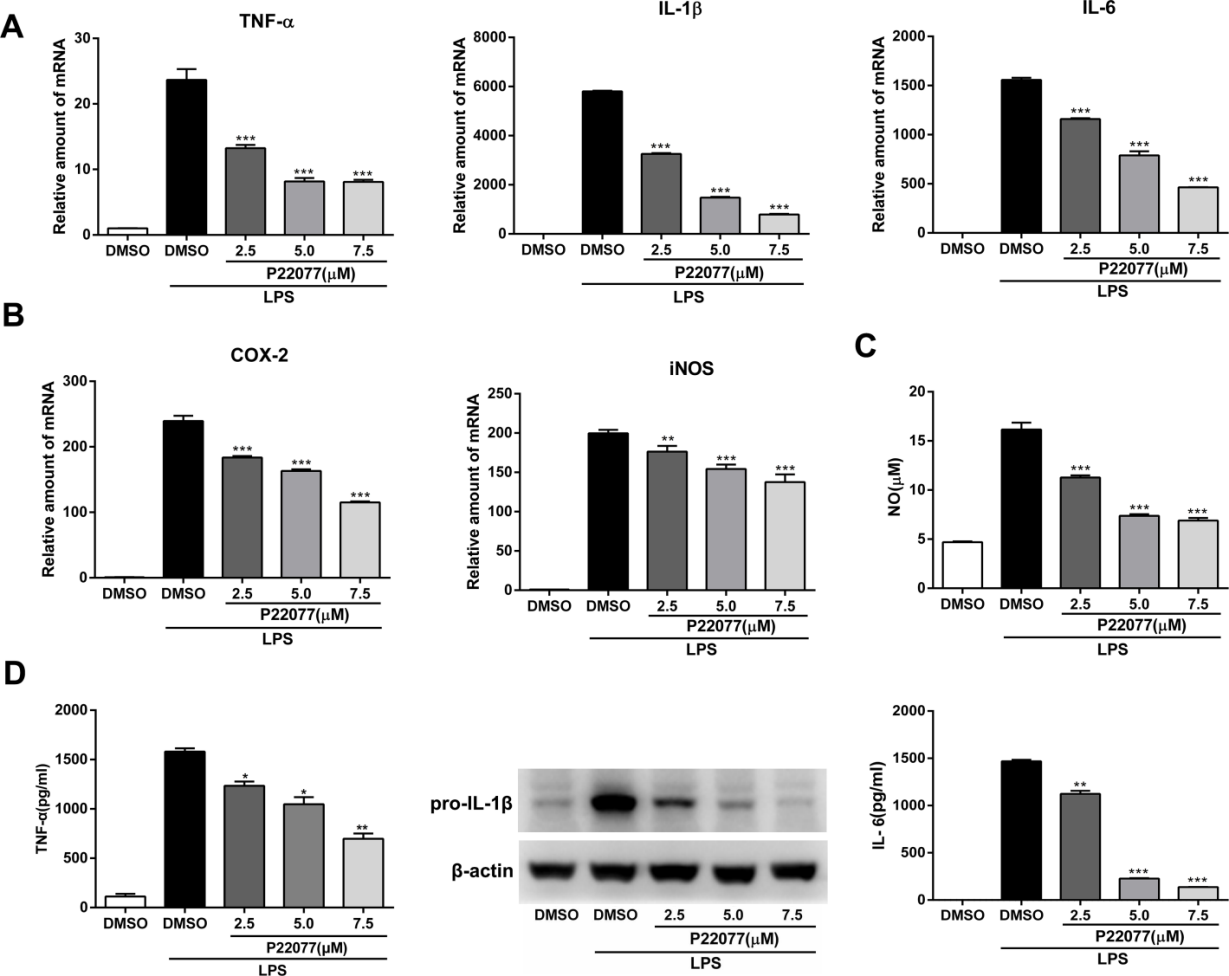
**P22077 suppresses LPS-induced inflammatory response in Raw264.7 cells.** (**A**, **B**) Raw264.7 cells were pretreated with DMSO or P22077 (2.5 μM, 5.0 μM and 7.5 μM) for 2 h, then stimulated with LPS (100 ng/ml) for another 4 h. The mRNA expressions of TNF-α, IL-1β, IL-6 (**A**), COX2 and iNOS (**B**) were analyzed by Q-PCR. (**C**, **D**) Raw264.7 cells were pretreated with DMSO or P22077 (2.5 μM, 5.0 μM and 7.5 μM) for 2 h, and then stimulated with LPS (100 ng/ml) for 10 h. Nitric oxide (NO) concentration in culture supernatant was measured by Griess assay (**C**). TNF-α and IL-6 in the supernatants were measured by ELISA, pro-IL-1β was detected by immunoblot (**D**). Similar results were obtained from three independent experiments and data were presented as mean ± SD of one representative experiment. *P<0.05, **P<0.01, ***P<0.001 vs LPS-stimulated DMSO group.

The expression of inflammatory mediators was a crucial response of macrophages with LPS stimulation. To investigate the anti-inflammatory effect of P22077 in macrophages, Raw264.7 cells were exposed to different concentrations of P22077 (2.5, 5.0 and 7.5 μM) and followed by LPS stimulation. The results showed that mRNA levels of TNF-α, IL-1β, IL-6, COX2 and iNOS were highly induced by LPS (100 ng/mL), and the expression of these pro-inflammatory cytokines were significantly decreased (up to 80%) in a dose-dependent manner with pretreated with P22077 (Figure 2A, 2B). Nitric oxide (NO) is a free radical, which is an important inflammatory signaling molecule. To examine the NO production, we use the Griess reagent to investigate the concentration of nitrite which is regard as biomarker of NO in supernatant. As expected, LPS obviously increased the release of NO and this effect could be inhibited by P22077 in a dose-dependent manner with maximum effects of about 50% NO reduction (7.5 μM P22077 compared with LPS alone group) (Figure 2C). Furthermore, we measured pro-inflammatory cytokines TNF-α and IL-6 in the cell culture supernatant by ELISA, and detected pro-IL-1β by immunoblot. The results showed that pretreated with P22077 suppressed the production of LPS-induced TNF-α, pro-IL-1β and IL-6 in Raw264.7 cells (Figure 2D). Taken together, these results indicate that P22077 exhibits anti-inflammatory properties in Raw264.7 cells.

### P22077 inhibits LPS-induced inflammatory response in mouse peritoneal macrophages

**Figure 3:**
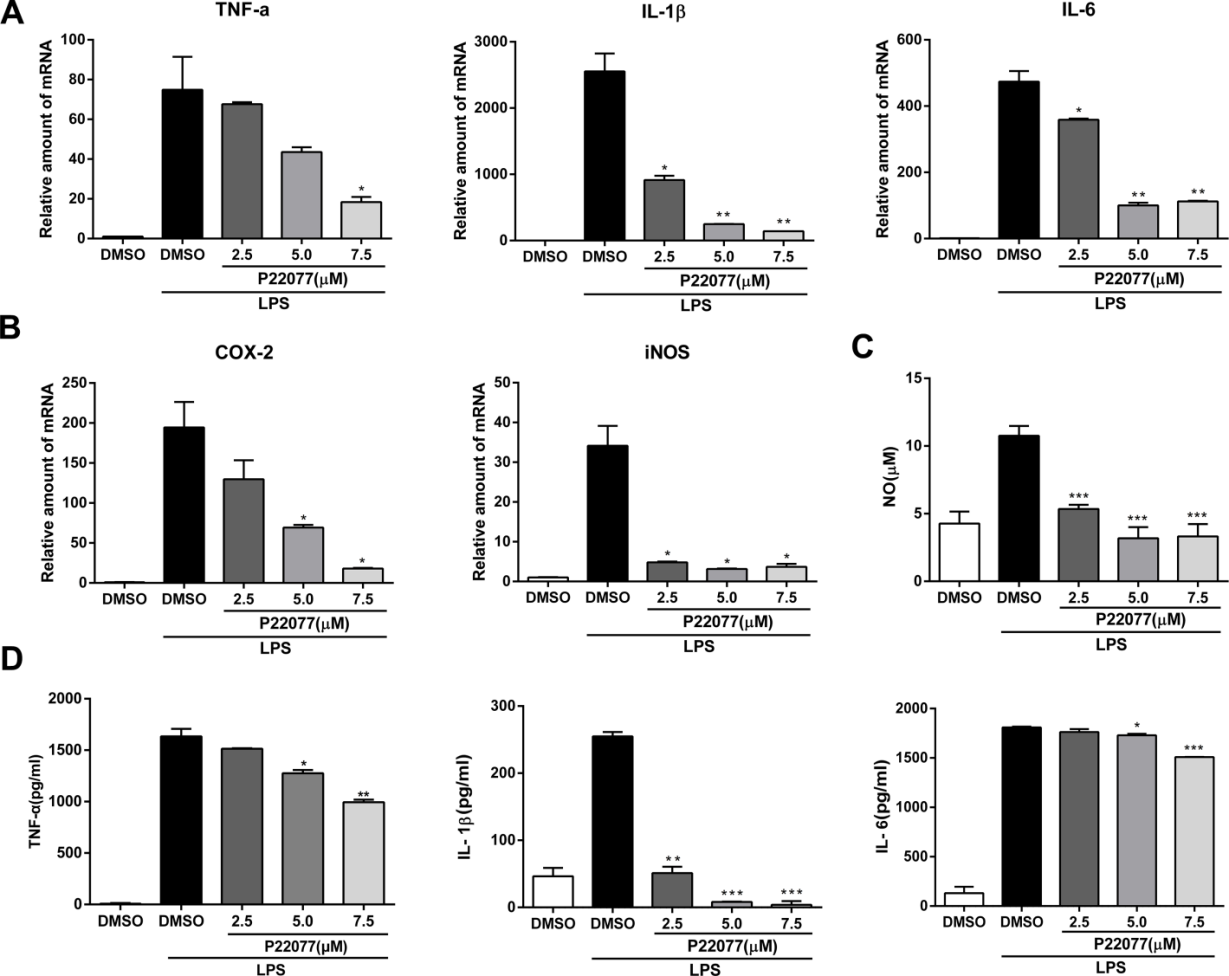
**P22077 suppresses LPS-induced inflammatory response in mouse peritoneal macrophages.** (**A**, **B**) Mouse peritoneal macrophages were pretreated with DMSO or P22077 (2.5 μM, 5.0 μM and 7.5 μM) for 2 h, then stimulated with LPS (100 ng/ml) for another 4 h. The mRNA expressions of TNF-α, IL-1β, IL-6 (**A**), COX2 and iNOS (**B**) were analyzed by Q-PCR. (**C**, **D**) Mouse peritoneal macrophages were pretreated with DMSO or P22077 (2.5 μM, 5.0 μM and 7.5 μM) for 2 h, and then stimulated with LPS (100 ng/ml) for 10 h. Nitric oxide (NO) concentration in culture supernatant was measured by Griess assay (**C**). TNF-α, IL-1β and IL-6 in the supernatants were measured by ELISA (**D**). Similar results were obtained from three independent experiments and data were presented as mean ± SD of one representative experiment. *P<0.05, **P<0.01, ***P<0.001 vs LPS-stimulated DMSO group.

To further confirm the anti-inflammatory effect of P22077 in a primary macrophages, we next studied the inflammatory response of P22077-treated mouse peritoneal macrophages. Q-PCR results showed that P22077 could significantly inhibit LPS-induced TNF-α, IL-1β, IL-6, COX2 and iNOS mRNA levels compared to control group, which is similar with that in Raw264.7 cells (Figure 3A, 3B). Besides, the production of NO, TNF-α, IL-1β and IL-6 in the cell culture supernatant was also significant inhibition by P22077 (Figure 3C, 3D). These results further confirm that P22077 has significant anti-inflammatory potential by suppressing LPS-induced inflammatory mediators production in mouse peritoneal macrophages.

### P22077 suppresses LPS-induced NF-κB and MAPKs signaling pathways activation

**Figure 4:**
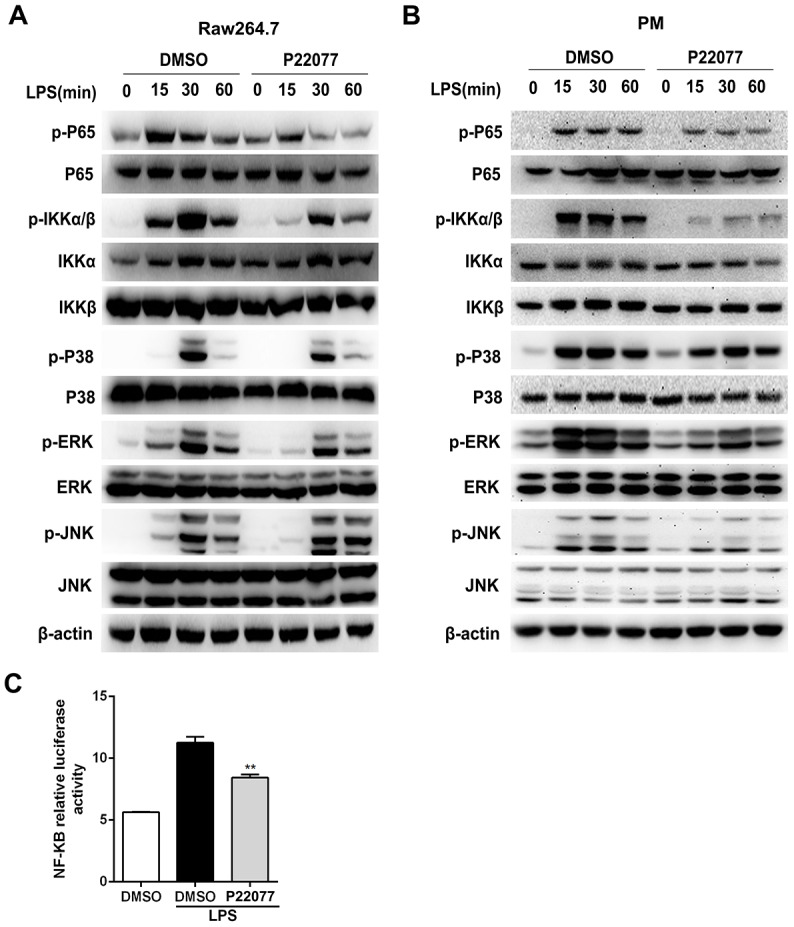
**P22077 inhibits the activation of NF-κB and MAPKs signaling pathways in LPS-stimulated Raw264.7 cells and mouse peritoneal macrophages.** (**A**) Raw264.7 cells were pretreated with DMSO and P22077 (7.5 μM) for 2 h, and then stimulated with LPS (100 ng/ml) for the indicated times and analyzed the indicated proteins by immunoblot. (**B**) Mouse peritoneal macrophages were pretreated with DMSO and P22077 (7.5 μM) for 2 h, and then stimulated with LPS (100 ng/ml) for the indicated times and analyzed the indicated proteins by immunoblot. (**C**) RAW 264.7 cells were transfected with NF-κB luciferase reporter plasmid and Renilla luciferase construct phRL-TK plasmid for 24 h and pretreated with the P22077 (7.5 μM) for 2 h before stimulation with 100ng/ml LPS for 4 h and luciferase activity was measured. Similar results were obtained from three independent experiments and data were presented as mean ± SD of one representative experiment. **P<0.01 vs LPS-stimulated group.

NF-κB and/or MAPKs signaling pathways play an important role in the regulation of innate immunity and inflammation. To determine whether NF-κB and/or MAPKs pathways were involved into the anti-inflammatory response of P22077, we examined the key phosphorylation proteins in NF-κB and MAPKs signaling pathways by immunoblot. The results showed that P22077 treatment inhibited LPS-induced p65 and IKKα/β phosphorylation in Raw264.7 cells (Figure 4A). Besides, P22077 also significantly inhibited LPS-induced phosphorylation of ERK and p38 in Raw264.7 cells (Figure 4A). Similarly, in mouse peritoneal macrophages, we also found that P22077 inhibited LPS-induced p65, IKKα/β phosphorylation in NF-κB signaling pathway and ERK, p38 phosphorylation in MAPKs signaling pathway (Figure 4B). Furthermore, the NF-κB luciferase reporter experiment also indicated that P22077 attenuated the activity of NF-κB signaling pathway (Figure 4C). Taken together, these findings reveal that P22077 suppresses LPS-induced inflammatory response by inhibiting the activation of NF-κB and MAPKs signaling pathways.

### P22077 promotes the degradation of TRAF6 in a proteasome-dependent manner

**Figure 5:**
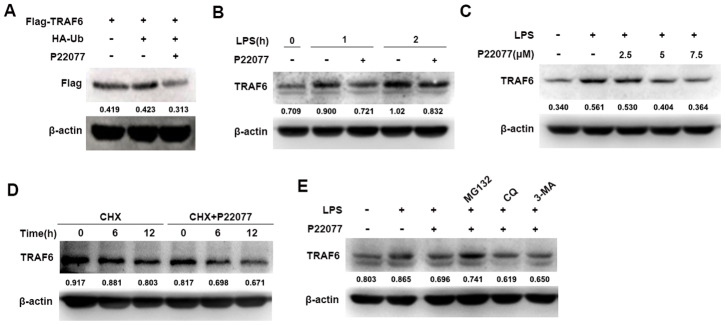
**P22077 promotes the degradation of TRAF6.** (**A**) HEK293T cells were transfected with Flag-TRAF6 and HA-Ub plasmid for 24h, and then treated with or without P22077 (7.5 μM) for 6 h, the Flag-TRAF6 protein was detected by immunoblot. (**B**) Mouse peritoneal macrophages were pretreated with or without P22077 (7.5 μM) for 2 h, and then stimulated with LPS (100 ng/mL) for 1 and 2 h. Immunoblot assay was used to analyze TRAF6 protein. (**C**) Mouse peritoneal macrophages were pretreated with various dose of P22077 (0, 2.5, 5 and 7.5 μM) for 2 h, and then stimulated with LPS (100 ng/mL) for 1 h. Immunoblot assay was used to analyze TRAF6 protein. (**D**) Mouse peritoneal macrophages were treated with cycloheximide (100 μg/ml) in the absence or presence of P22077 (7.5 μM) for 0, 6 and 12 h. Immunoblot assay was used to analyze TRAF6 protein. (**E**) Mouse peritoneal macrophages were pretreated with P22077 (7.5 μM), MG132 (20 μM), Chloroquine (50μM) or 3-MA (1 mM) for 2 h, and then stimulated with LPS (100 ng/mL) for 1 h. Immunoblot assay was used to analyze TRAF6 protein. Numbers below lanes indicated densitometry of the protein presented relative to β-actin. Similar results were obtained from three independent experiments and data were presented of one representative experiment.

To fully understand the function of P22077 in the NF-κB signaling pathway, further study was performed to identify the molecular target of P22077. NF-κB signaling pathway was complicated, its upstream protein include TRAF6, TAK1 and other signaling proteins. As previously reported, TRAF6 is a key adaptor molecular for the activation of the NF-κB signaling pathway and USP7 is an important deubiquitinase which stabilizes TRAF6 protein. Therefore, we speculated that P22077 inhibited USP7 protein activity, and thus promoted TRAF6 degradation on upstream of NF-κB signaling pathway. To verify this hypothesis, we over-expressed Flag-TRAF6 in HEK293T cells and then treated with or without P22077, we found that P22077 obviously reduced the protein level of TRAF6 (Figure 5A). Furthermore, Western blot analysis showed that P22077 treatment decreased the levels of TRAF6 in LPS-stimulated mouse peritoneal macrophages (Figure 5B). In addition, the quantity of TRAF6 was reduced by treated P22077 in a dose-dependent manner (Figure 5C). We treated mouse peritoneal macrophages with CHX (an inhibitor of protein synthesis) in order to explore whether P22077 affecting TRAF6 synthesis or degradation, and we found P22077 could increase TRAF6 degradation (Figure 5D). Using different protein degradation inhibitors such as MG132, CQ and 3-MA, we confirmed that P22077 promoted TRAF6 degradation in a proteasome-dependent manner as only MG132 rescuing the degradation of TRAF6 (Figure 5E). These results indicate that P22077 promotes the degradation of TRAF6 in a proteasome-dependent manner.

### P22077 promotes K48-linked ubiquitination of TRAF6

**Figure 6:**
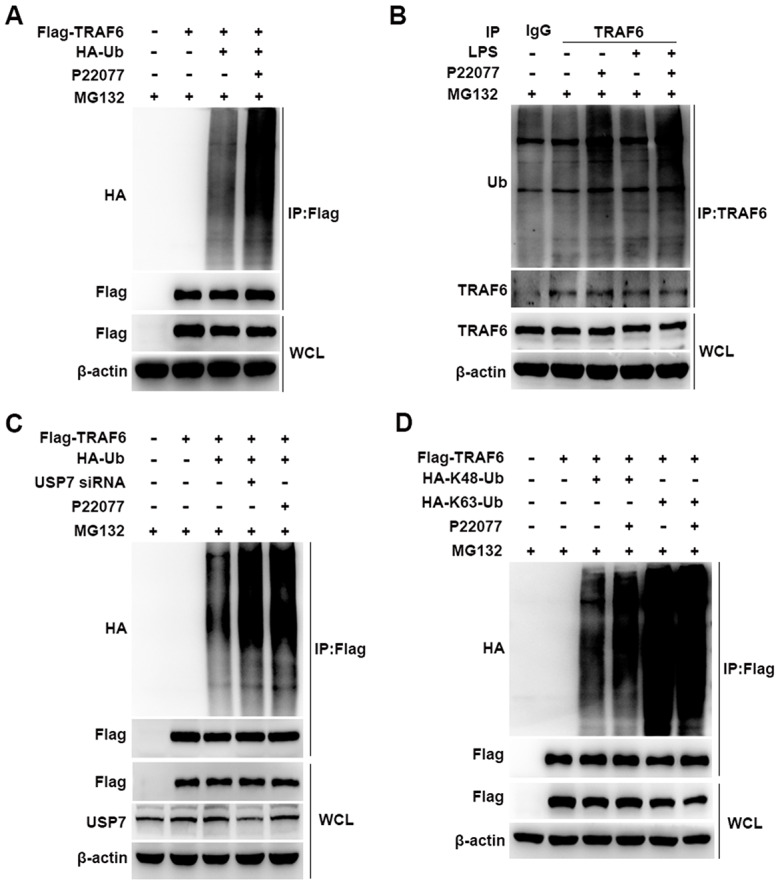
**P22077 promotes the K48-linked ubiquitination of TRAF6.** (**A**) HEK293T cells were transfected with Flag-TRAF6 and HA-Ub plasmid for 24h and then treated with P22077 (7.5 μM) and MG132 (20 μM) for 6 h, immunoprecipitation assay was used to detect ubiquitination level of TRAF6. (**B**) Mouse peritoneal macrophages were pretreated with P22077 (7.5 μM) and MG132 (20 μM) for 2 h, and then stimulated with LPS (100 ng/mL) for 1 h. Immunoprecipitation assay was used to analysis of TRAF6 protein ubiquitination level. (**C**) HEK293T cells were knockdown USP7, and transfected with Flag-TRAF6, HA-Ub, and then treated with P22077 (7.5 μM) and MG132 (20 μM) for 6 h, immunoprecipitation assay was used to detect the ubiquitination level of TRAF6. (**D**) HEK293T cells were transfected with Flag-TRAF6, HA-K48-Ub and HA-K63-Ub plasmid for 24h and then treated with P22077 (7.5 μM) and MG132 (20 μM) for 6 h, immunoprecipitation assay was used to detect the K48 and K63 sites of TRAF6 ubiquitination. Similar results were obtained from three independent experiments and data were presented of one representative experiment.

Having shown that P22077 promotes TRAF6 degradation in a proteasome- dependent manner, we further studied the cellular function of P22077 toward TRAF6. Flag-TRAF6 plasmid and HA-Ub plasmid were co-transfected into HEK293T cells with following MG132 and P22077 treatment. We found that P22077 significantly increased the ubiquitination level of TRAF6 (Figure 6A). In mouse peritoneal macrophages, we also confirmed these results. Ubiquitination assay showed that TRAF6 ubiquitination was significantly increased in mouse peritoneal macrophages pretreated with P22077 (Figure 6B). In addition, the ubiquitination level of TRAF6 was elevated both in P22077 treated and USP7 knockdown cells (Figure 6C). Subsequently, to study the form of ubiquitination chains linking to TRAF6, HEK293T cells were transfected with Lys-mutated ubiquitin plasmids (K48 or K63). The mutated K48 or K63 sites ubiquitin has only one lysine in ubiquitin at position 48 or 63, respectively. Ubiquitination assay demonstrated that P22077 significantly increased K48-linked but not K63-linked ubiquitination of TRAF6 (Figure 6D). Taken together, these data indicate that P22077 promotes K48-linked polyubiquitination-dependent degradation of TRAF6.

### P22077 relieves LPS-induced inflammatory response *in vivo*

**Figure 7:**
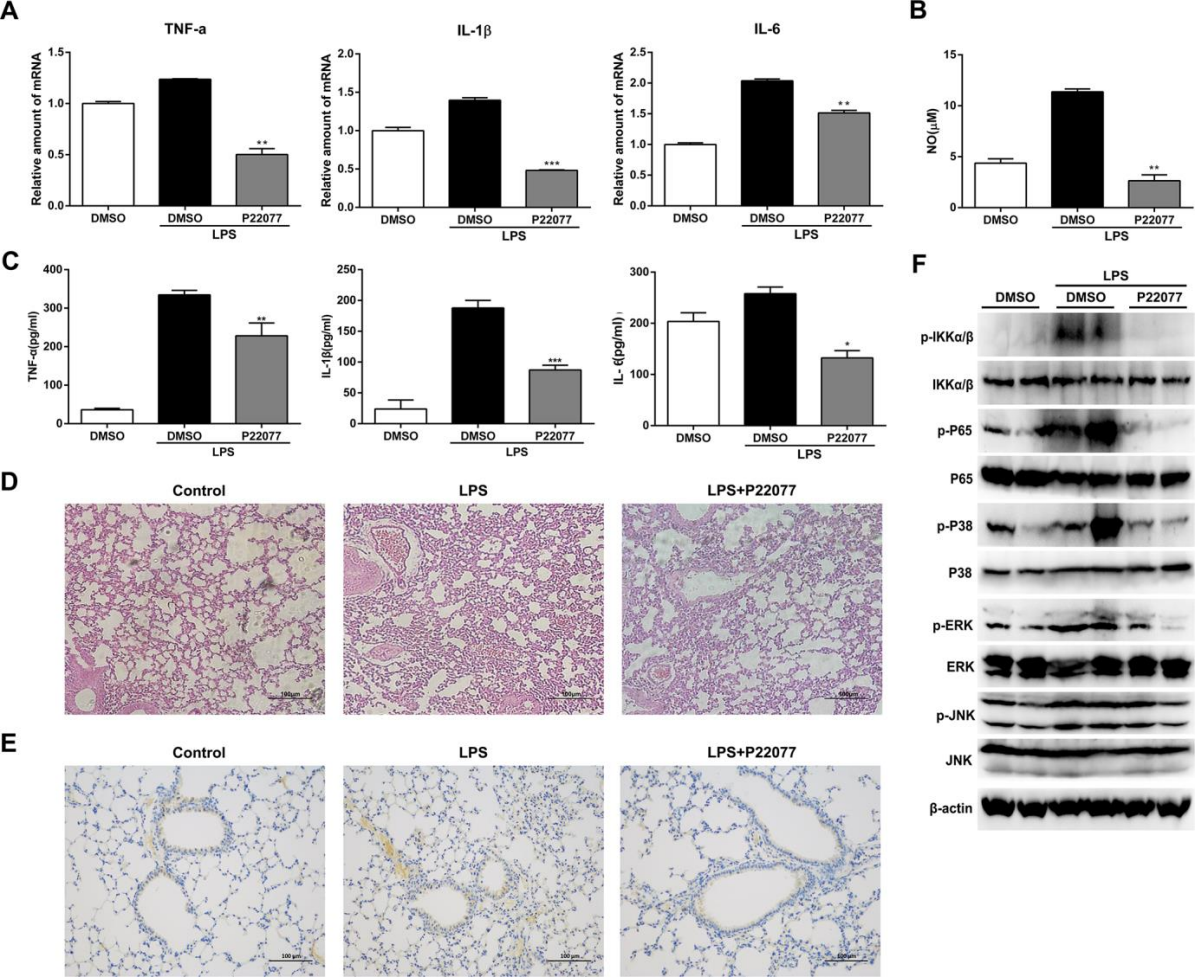
**P22077 relieves LPS-induced inflammatory response in mice.** C57BL/6 mice (10 mice/group) were intraperitoneally injected with DMSO or P22077 (15 mg/kg), following challenged with PBS or LPS (10 mg/kg). Mice were sacrificed after 2 h. (**A**) TNF-α, IL-1β and IL-6 mRNA level in lung tissue were analyzed by Q-PCR. (**B**) Nitric oxide (NO) concentrations in the serum were examined by Griess assay. (**C**) TNF-α, IL-1β and IL-6 concentrations in the serum were examined by ELISA. (**D**) Left lung was collected for HE staining, and representative images were displayed. Scale bars, 100 μm. (**E**) Left lung was collected for immunohistochemistry staining of TRAF6. Scale bars, 100 μm. (**F**) Lung tissue was grinded and centrifuged, and the supernatant was used to analyzed the indicated proteins by immunoblot. Similar results were obtained from three independent experiments and data were presented as mean ± SD of one representative experiment. *P<0.05, **P<0.01, ***P<0.001 vs LPS-stimulated DMSO group (**A**–**C**).

To investigate the anti-inflammatory properties of P22077 *in vivo*, we induced a systemic acute inflammation mice model by LPS intraperitoneal injection [30–32]. It is characterized by overproduction of inflammatory cytokines and leads to the lethal multiple organ damage after LPS stimulation. Before LPS challenge, C57BL/6 mice were intraperitoneally injected with P22077 (15 mg/kg) or vehicle DMSO. Two hours after LPS challenge, mice were sacrificed to obtain serum and lung. As shown in Figure 7A, P22077 treatment suppressed mRNA expression of pro-inflammatory cytokines such as TNF-α, IL-1β and IL-6 in lung tissue. In addition, compared to control group, the serum levels of NO, TNF-α, IL-1β and IL-6 were decreased in the mice treated with P22077 (Figure 7B–7C). H&E staining of lung tissue showed that P22077 treatment reduced LPS-induced pulmonary interstitial edema and inflammatory infiltrates (Figure 7D). Immunohistochemistry staining of TRAF6 in lung tissue suggested that LPS induced the expression of TRAF6, but when treated with P22077, the protein of TRAF6 was significantly reduced (Figure 7E). We also examined the key phosphorylation proteins in NF-κB and MAPKs signaling pathways in lung tissue, the results suggested that the phosphorylation of IKKα/β, p65 in NF-κB signaling pathway, and p38, ERK in MAPKs signaling pathway were significantly decreased in P22077 treated mice, as observed in Raw264.7 cells and mouse peritoneal macrophages (Figure 7F). These data indicate that P22077-treated mice were more resistant to LPS-induced inflammation and lung injury.

## DISCUSSION

**Figure 8:**
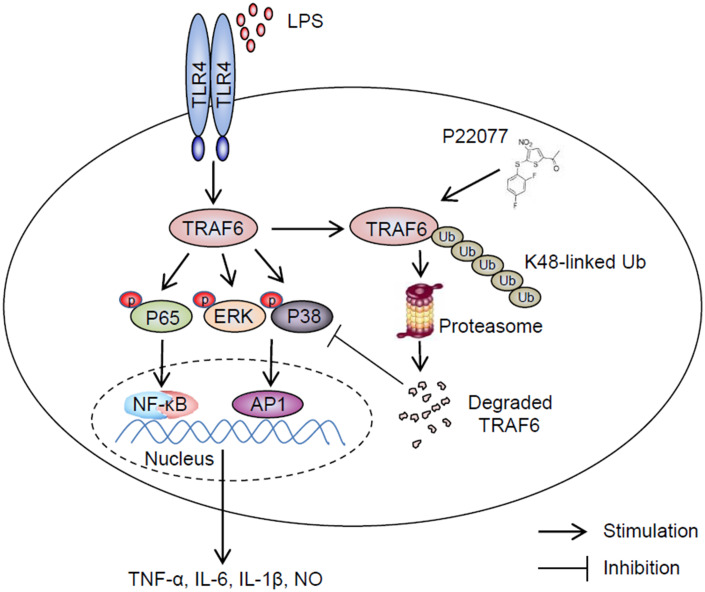
**Schematic of the anti-inflammatory mechanism of P22077.** P22077 promotes K48-linked ubiquitination and degradation of TRAF6, and subsequently inhibits the LPS-induced NF-κB and MAPKs pathway to alleviate inflammation.

It is still a challenge for human to overcome inflammatory disease which due to invading of exogenous pathogens, trauma and some unknown reason, particularly some widespread chronic diseases such as Alzheimer [33, 34]. Developing more effective and less toxic drugs to treat inflammatory diseases is extremely urgent. Up to now, larger number of strategies have been adopted, including reduce the activity of specific cytokines or their receptors, block cell adhesion and migration, deplete B or T lymphocytes, etc. Among different strategies, identifying small-molecule inhibitors targeting downstream of cytokine receptors to reduce production or activities of pro-inflammatory cytokines was regard as a promising method [35–37]. Post-translational modifications, such as ubiquitination and deubiquitination played a crucial role in signaling cascades. Furthermore, deubiquitination effect of some DUBs, such as A20 [38], CYLD [39, 40] and OTULIN (also known as FAM105B) [41], were found playing a critical role in inflammatory response induced by TNF-α, lipopolysaccharide (LPS) and some other stimulus. All of these imply ubiquitin proteasome system (UPS) was a potential drug targets in anti-inflammatory process. In this study, we demonstrated P22077, as an inhibitor of USP7, was a potential anti-inflammatory drug that inhibited secretion of pro-inflammatory cytokines by promoting K48-linked ubiquitination and degradation of TRAF6 (Figure 8). In our study, P22077 did not appear to impact the K63-linked ubiquitination of TRAF6. But Xiang et al. reported that USP7 regulated the K63-linked polyubiquitination of TRAF3 and TRAF6 [42], indicated that USP7 regulating polyubiquitination of TRAF6 maybe difference in different stimulus. Furthermore, our data suggest that small molecule inhibitor of DUBs maybe an excellent anti-inflammatory drug with potential clinical application.

Bacterial lipopolysaccharides (LPS) consist of lipid A was a typical ligand of plasma membrane protein TLR4, which can induce host innate immune response [43]. Macrophages are key cells for inflammatory processes which involved in all phases of inflammation, regulating acute inflammatory response and chronic pathological changes [44]. In this study, we utilized two different kinds of macrophages stimulated by LPS to investigate the roles of P22077 in inflammation process. Pro-inflammatory cytokines (TNF-α, IL-1β, and IL-6), Cyclooxygenase-2 (COX-2), inducible nitric oxide synthase (iNOS) increase dramatically over the following several hours stimulated with LPS that are regard as biomarkers of inflammatory processes [45]. In addition, nitric oxide (NO) is characteristic of acute inflammation for inhibiting leukocyte adhesion to vascular endothelium [46]. Here we provided sufficient and systemic evidence on mRNA and protein level exhibiting that P22077 inhibits LPS-induced production of pro-inflammatory cytokines or mediators (TNF-α, IL-1β, IL-6, COX-2, iNOS and NO) not only in Raw264.7 cells and mouse peritoneal macrophages, but also in C57BL/6 mice. The results of lung HE staining from mice potently supported our finding. Our data confirmed that P22077 could reduce the secretion of inflammatory mediators and relieved inflammatory response *in vitro* and *in vivo*.

LPS recognized by TLR4, and then a lot of molecules participate in signaling cascade, including MyD88, TRAF, TAK1 and several downstream kinases. Among them, NF-κB and MAPKs signaling pathways were two critical pathways. Here, we detected some key signaling proteins in these two signaling pathways, and phosphorylation of NF-κB was significantly down-regulated both in Raw264.7 cells and mouse peritoneal macrophages after treatment with P22077. Moreover, NF-κB luciferase reporter gene assay also confirmed P22077 certainly inhibited NF-κB signaling pathway activation. Li et al. found that USP7 downregulated NF-κB signaling by interacting with HSCARG [23], but Colleran et al. found USP7 promoted inflammation by deubiquitination of NF-κB [22]. Based on these findings of two groups above, we have reasons to believe that USP7 affects inflammatory response with a variety of mechanisms. Furthermore, it has been reported that decreasing of USP7 expression coincided with reducing cellular TRAF6 protein [24]. Additionally, A20 also been reported participating in suppressing Toll-like receptor–induced activity of NF-κB signaling and pro-inflammatory gene expression in macrophages by removing ubiquitin chains from TRAF6 [47]. Recently, study showed that resveratrol could suppress NF-κB activation by attenuating TRAF6 expression and ubiquitination [48, 49]. So, we assumed that P22077 negatively regulated inflammation by targeting of TRAF6. Next, we verified that P22077 promoted the degradation of TRAF6 in a proteasome-dependent manner. Our study basically confirmed that P22077 could inhibit activation of NF-κB and MAPKs signaling pathways by promoting K48-linked ubiquitination and degradation of TRAF6.

In conclusion, we demonstrate that P22077 can promote K48-linked ubiquitination and degradation of TRAF6 and then inhibit the activation of NF-κB and MAPKs signaling pathways, and then negatively regulates inflammatory response. These findings provide a new insight into the small-molecule compounds for inhibition of NF-κB and MAPKs-mediated inflammation and provides a new potential anti-inflammatory drug to cure infection-related inflammatory and autoimmune diseases.

## MATERIALS AND METHODS

### Reagents

P22077 was purchased from SelleckChem. Phospho-MAPK Family Antibody Sampler Kit, NF-κB Pathway Sampler Kit, pro-IL-1β antibody and ubiquitin antibody were purchased from Cell Signaling Technology. TRAF6 antibody was purchased from Abcam. Flag and HA tag antibodies were purchased from GenScript. β-actin antibody was purchased from HuaAn Biotechnology. DMSO, cyclohexane and LPS were obtained from Sigma-Aldrich.

### Cells and mice

6-8 weeks C57BL/6 female mice (16–25g) were purchased from Joint Ventures Sipper BK Experimental Animals. Mice were feed in pathogen-free condition with 12 h light and 12 h darkness circulation. All animal experiments carried out according to the National Institute of Health Guide for the Care and Use of Laboratory Animals, and experiment protocols were permitted by the Shenzhen University, Shenzhen. Mouse peritoneal macrophages were obtained from C57BL/6 mice after 4 days following injection of 2 ml of 3% thioglycolate broth and isolated adhered cells after 3 h. The mouse peritoneal macrophages were cultured in RPMI 1640 with 10% fetal bovine serum. Raw264.7 cells and HEK-293T cells were from ATCC and cultured in DMEM medium supplemented with 10% fetal bovine serum.

### Cell viability assay

Cells equivalent to 1×10^4^/well were plated in 96-well plates overnight, and then stimulated by different concentrations of P22077 for 12 h. Cell viability was assessed using MTT assay [3-(4,5)-dimethyl thiazol-2,5-diphenyl tetrazolium bromide] as reagent supplies manual described (Beyotime Biotechnology).

### Plasmid transfection

2 × 10^6^ 293T cells were seeded into 6-cm dish and cultivated overnight. 293T cells were transfected with plasmid using JetPEI (Polyplus) according to the manufacturer's instructions. Culture medium was changed 12 h after transfection. Cells were further treated and collected for subsequent experiments.

### Quantitative PCR

Total cellular RNA was extracted by Trizol reagent and measured expression of mRNA. Gene-specific primer sequences were as following: mus β-actin forward, 5’-AGTGTGACGTTGACATCCGT-3, and reverse, 5’- GCAGCTCAGTAACAGTCCGC-3; mus TNF-α forward, 5’- AAGCCTGTAGCCCACGTCGTA-3, and reverse, 5’-GGCACCACTAGTTGGTTGTCTTTG-3; mus IL-1β forward, 5’-AACTGTTCCTGAACTCAACTGT-3, and reverse, 5’- GAGATTTGAAGCTGGATGCTCT-3; mus IL-6 forward, 5’- TAGTCCTTCCTACCCCAATTTCC-3, and reverse, 5’- TTGGTCCTTAGCCACTCCTTC-3; mus iNOS forward, 5’- CAGCACAGGAAATGTTTCAGC-3, and reverse, 5’- TAGCCAGCGTACCGGATGA-3; mus COX2 forward, 5’- CCCTGAAGCCGTACACATCA-3, and reverse, 5’- TGTCACTGTAGAGGGCTTTCAATT-3.

### Immunoblotting and ubiquitination assay

293T cells, Raw264.7 cells or mouse peritoneal macrophages were lysis by NP40 lysis buffer containing 1×protease inhibitor mixture, centrifuge for 5 min at 12,000g. cell supernatants were collected and added 1×loading buffer followed boiling 5min. equivalent amounts of protein were subjected to 8%–15% SDS-PAGE and transferred onto PVDF membranes. Membranes were blocked in 5% skim milk, and incubated overnight with primary antibodies at 4°C followed by incubation with secondary antibodies for 1 h at room temperature. Proteins on membranes were visualized using the Chemiluminescent Reagents Kit (Thermo Scientific). Signals were detected with the FluorChem E (Cell biosciences).

For ubiquitination assay, cells were lysed in immunoprecipitation buffer (with 1% SDS) containing protease inhibitor mixture and boiled for 5min at 95 °C. Supernatant was collected and diluted 10-fold in immunoprecipitation buffer, following immune-precipitation with specific antibody for 2 hours, and then incubated with protein A/G Plus-Agarose Immunoprecipitation reagent (Santa Cruz Biotechnology) for 6 hours, beads was washed five times with immunoprecipitation buffer. Protein samples were boiled with SDS sample buffer 5 minutes and equal amounts of protein were used to 8%-12% SDS-PAGE and transferred into nitrocellulose membranes, immunoblot used specific antibodies.

### Enzyme-linked immunosorbent assay (ELISA)

RAW264.7 cells or mouse peritoneal macrophages were treated with LPS with or without P22077. Then, cell culture medium was collected and centrifuged at 4 °C, 16000 rpm for 10 min. The supernatants were collected and used for detecting TNF-α, IL-6 or IL-1β concentrations by ELISA according to the manufacturer's instructions (Invitrogen).

### Measurement of nitric oxide (NO)

Nitric oxide levels in cell culture supernatants were quantified by Griess Assay as reagent supplies manual described (Promega).

### NF-κB luciferase reporter gene experiment

293T cells were co-transfected with 90 ng of NF-κB luciferase reporter plasmid and 10 ng of Renilla luciferase construct phRL-TK (Promega) according to supplies manual. 24 h after transfection, cells were pretreated with DMSO or P22077 for 2 h, followed stimulation with LPS (100 ng/ml) for 30min. The relative luciferase activity was calculated by dividing the Firefly luciferase activity by the Renilla luciferase activity.

### Statistical analysis

All data were presented as the mean ± SD, and at least three independent experiments were performed. Results were statistically analyzed by Student´s t-test, and analyses were performed using GraphPad Prism version 6.0 software, with statistical significance set at p < 0.05.
